# Impact Monitoring of the National Scale Up of Zinc Treatment for Childhood Diarrhea in Bangladesh: Repeat Ecologic Surveys

**DOI:** 10.1371/journal.pmed.1000175

**Published:** 2009-11-03

**Authors:** Charles P. Larson, Unnati Rani Saha, Hazera Nazrul

**Affiliations:** 1 International Centre for Diarrhoeal Disease Research, Bangladesh (ICDDR,B), Dhaka, Bangladesh; 2 Centre for International Child Health, British Columbia Children's Hospital and Department of Pediatrics, University of British Columbia, Vancouver, Canada; Aga Khan University, Pakistan

## Abstract

**Background:**

Zinc treatment of childhood diarrhea has the potential to save 400,000 under-five lives per year in lesser developed countries. In 2004 the World Health Organization (WHO)/UNICEF revised their clinical management of childhood diarrhea guidelines to include zinc. The aim of this study was to monitor the impact of the first national campaign to scale up zinc treatment of childhood diarrhea in Bangladesh.

**Methods/Findings:**

Between September 2006 to October 2008 seven repeated ecologic surveys were carried out in four representative population strata: mega-city urban slum and urban nonslum, municipal, and rural. Households of approximately 3,200 children with an active or recent case of diarrhea were enrolled in each survey round. Caretaker awareness of zinc as a treatment for childhood diarrhea by 10 mo following the mass media launch was attained in 90%, 74%, 66%, and 50% of urban nonslum, municipal, urban slum, and rural populations, respectively. By 23 mo into the campaign, approximately 25% of urban nonslum, 20% of municipal and urban slum, and 10% of rural under-five children were receiving zinc for the treatment of diarrhea. The scale-up campaign had no adverse effect on the use of oral rehydration salt (ORS).

**Conclusions:**

Long-term monitoring of scale-up programs identifies important gaps in coverage and provides the information necessary to document that intended outcomes are being attained and unintended consequences avoided. The scale-up of zinc treatment of childhood diarrhea rapidly attained widespread awareness, but actual use has lagged behind. Disparities in zinc coverage favoring higher income, urban households were identified, but these were gradually diminished over the two years of follow-up monitoring. The scale up campaign has not had any adverse effect on the use of ORS.

*Please see later in the article for the Editors' Summary*

## Abstract 2

Charles Larson and colleagues find that 23 months into a national campaign to scale up zinc treatment for diarrhea in children under age 5 years, only 10% of children with diarrhea in rural areas and 20%–25% in urban/municipal areas were getting the treatment.

## Editors' Summary

**Background:**

Diarrheal disease is a significant global health problem with approximately 4 billion cases and 2.5 million deaths annually. The overwhelming majority of cases are in developing countries where there is a particularly high death rate among children under five years of age. Diarrhea is caused by bacterial, parasitic, or viral pathogens, which often spread in contaminated water. Poor hygiene and sanitation, malnutrition, and lack of medical care all contribute to the burden of this disease. Replacing lost fluids and salts is a cheap and effective method to rehydrate people following dehydration caused by diarrhea. Clinical trials show that zinc, as part of a treatment for childhood diarrhea, not only helps to reduce the severity and duration of diarrhea but also reduces the likelihood of a repeat episode in the future. Zinc is now included in the guidelines by the World Health Organization (WHO)/UNICEF for treatment of childhood diarrhea.

**Why Was This Study Done?:**

Zinc treatment together with traditional oral rehydration salts therapy following episodes of diarrhea could potentially benefit millions of children in areas where diarrheal disease is prevalent. The “Scaling Up of Zinc for Young Children” (SUZY) project was established in 2003 to provide zinc treatment for diarrhea in all children under five years of age in Bangladesh. The project was supported by a partnership of public, private, nongovernmental organization, and multinational sector agencies during its scale up to a national campaign across Bangladesh. The partners helped to develop the scale-up campaign, produce and distribute zinc tablets, train health professionals to provide zinc treatment, and create media campaigns (such as advertisements in TV, radio, and newspapers) to raise awareness and promote the use of zinc for diarrhea. The researchers wanted to monitor how effective and successful the national campaign was at promoting zinc treatment for childhood diarrhea. Also, they wanted to highlight any potential problems during the implementation of health care initiatives in areas with deprived health systems.

**What Did the Researchers Do and Find?:**

The researchers set up survey sites to monitor results from the first two years of the SUZY campaign. Four areas, each representing different segments of the population across Bangladesh were surveyed; urban slums, urban nonslums, municipal (small city), and rural. There are approximately 1.5 million children under the age of five across these sites. Households in each survey site were selected at random, and seven surveys were conducted at each site between September 2006 and October 2008—about 3,200 children with diarrhea for each survey. Over 90% of parents used private sector providers of drug treatment so the campaign focused on distribution of zinc tablets in the private sector. They were also available free of charge in the public health sector. TV and radio campaigns for zinc treatment rapidly raised awareness across Bangladesh. Awareness was less than 10% in all communities prelaunch and peaked 10 months later at 90%, 74%, 66%, and 50% in urban nonslum, municipal, urban slum, and rural sites, respectively. However, after 23 months only 25% of urban nonslum, 20% of municipal and urban slum, and 10% of rural children under five years of age were actually using zinc for childhood diarrhea. Use of zinc was shown to be safe, with few side-effects, and did not affect the use of traditional treatments for diarrhea. Researchers also found that many children were not given the correct ten-day course of treatment; 50% of parents were sold seven or fewer zinc tablets.

**What Do These Findings Mean?:**

These findings show that the first national campaign promoting zinc treatment for childhood diarrhea in Bangladesh has had some success. Addition of zinc tablets for diarrhea treatment did not interfere with existing therapies. Mass media campaigns, using TV and radio, were useful for promoting health care initiatives nationwide alongside the education of health care providers and care-givers. The study also identified areas where more work is needed. Surveys in more remote, hard to reach sites in Bangladesh would provide better representation of the country as a whole. High awareness of zinc did not translate into high use. Repeated surveying in the same subdistricts may have overestimated actual awareness levels. Furthermore, mass media messages must link with messages from health care providers to help to reinforce and promote understanding of the use of zinc. A change in focus of media messages from awareness to promoting household decision-making may aid the adoption of zinc treatment for childhood diarrhea and improve adherence.

**Additional Information:**

Please access these Web sites via the online version of this summary at http://dx.doi.org/10.1371/journal.pmed.1000175

- The International Centre for Diarrhoeal Disease Research, Bangladesh Web site has information about the study
- The World Health Organisation provides information on diarrhea
- The study was sponsored by the Bill & Melinda Gates Foundation

## Introduction

About 1.9 million children under the age of 5 y annually die from diarrhea, accounting for 19% of all under-five mortality [1]. Clinical trials of zinc treatment in children 6 mo to 5 y of age have consistently demonstrated its ability to reduce disease duration and severity as well as the likelihood of a repeat episode [2]–[4]. It has been estimated that the successful scaling up of zinc treatment for childhood diarrhea could potentially save 400,000 under-five deaths per year [5]. In response to the curative and preventive evidence in support of zinc treatment, in 2004 WHO/UNICEF revised their clinical management of childhood diarrhea guidelines to include zinc treatment of any episode [6]. The present challenge is the scale up of zinc treatment and other life-saving interventions within resource-deprived health systems with limited capacity to absorb additional services. As has been pointed out by others, efforts to bring zinc treatment to scale have the potential to significantly benefit children, but may also have harmful consequences through their impact on other health behaviours or services [7],[8].

The Scaling Up of Zinc for Young Children (SUZY) Project was established in 2003 with the aim of setting Bangladesh on the path to covering all under-five children with zinc treatment of any diarrheal illness episode. A partnership was created that included public, private, nongovernmental organisation, and multinational sector agencies. Over a period of 3 y activities in support of preparing for the national scale up included formative and operational research, product registration and technology transfer, awareness building and orientation of health professionals, and preparation of mass media messages. In December, 2006 a national mass media campaign to promote a dispersible tablet zinc formulation, “Baby Zinc,” for the treatment of childhood diarrhea was launched. All media messages linked zinc treatment to the continued use of oral rehydration salts (ORS).

In order to monitor the success of the project in achieving its intended aims, as well as other unintended consequences, continuous nationally representative zinc coverage surveys have been carried out in Bangladesh that coincide with the national launch of a mass media zinc treatment promotion campaign. This article describes the results of the national scale-up campaign over the initial 2 y of its conduct. The primary outcomes of interest were the documentation of the proportion of children receiving zinc treatment for a diarrheal illness, identifying disparities in zinc coverage, and monitoring for potential unintended outcomes, in particular decreased use of ORS.

## Methods

This study was reviewed and approved by the Research Review and Ethical Review Committees of the International Centre for Diarrhoeal Disease Research, Bangladesh (ICDDR,B). Given the high levels of suspicion created when asked to sign a document they cannot read and the minimal potential for harm, the Ethics Review Committee of ICDDR,B approved an informed verbal consent. A verbal consent was obtained from all participating interviewees, with the interviewer required to sign that consent when obtained (Text S2).

### Study Design

Repeat ecologic surveys were carried out in four representative population strata: urban Dhaka (mega-city) slum and nonslum, municipal (small city), and rural. Prior to the launch of the planned mass media campaign a baseline survey was completed between September and November 2006. Thereafter, postlaunch surveys were repeated over the following dates; 12/2006 to 02/2007 (1–3 mo), 03 to 05/2007 (4–6 mo), 06 to 09/2007 (7–10 mo), 10/2007 to 01/2008 (11–14 mo), 02 to 05/2008 (15–18 mo), and 06 to 10/2008 (19–23 mo).

### Population

#### Source population

**Figure 1:**
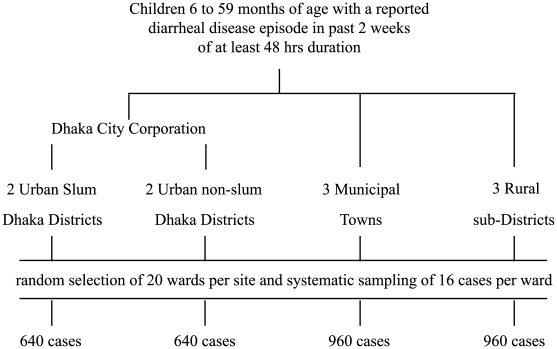
Summary of the cluster sampling framework for the surveys.

There are two large “mega-cities” in Bangladesh, one of which, Dhaka, was arbitrarily chosen (Figure 1). In Dhaka all districts were stratified as either predominantly urban slum or nonslum populations and two districts from each category were randomly selected. Rural subdistricts were purposively selected on the basis of their proximity to ICDDR,B field sites in the west (Abhonagar), southeast (Mirsarai), and northeast (Hobiganj) of Bangladesh. The municipal district capitals representing each rural site were also selected (Khuna, Camila, and Sylhet). The under-five population of these ten sites is approximately 1.5 million children.

#### Study population

Within the selected sites, all wards (defined as urban census tracts or an administrative grouping of two to three villages) were listed and 20 randomly selected prior to the start of each survey round. Standard WHO EPI (Expanded Program for Immunization) cluster methods were applied [9]. These methods eliminate the need to enumerate and then randomly select households. In rural settings a site central to the village was chosen and a wheel board spun to determine the direction of households to be visited following a more or less straight line. Upon reaching the peripheral boundary of the village the interviewers returned to the central point and spun the wheel again. Within urban wards a central starting point was chosen and then a systematic, door-to-door survey of households was carried out. Within each rural or urban ward the survey was stopped once 16 children, 6–59 mo of age with an active or recent (within the past 2 wk) episode of diarrhea of at least 48-h duration had been identified, and informed verbal consent from either parent obtained. If more than one child in the household was eligible, one was randomly chosen.

#### Sample size estimation

Setting the level of confidence at 0.95 and a minimal detectable error of 0.05 around a prevalence estimate, assuming the overall prevalence of zinc use to be 0.20 and adjusting by 2.5 for design effect (DE), a minimum of 615 cases per population strata were estimated to be required. The 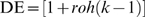, where *roh* (rate of homogeneity) was estimated to be 0.10 and *k* was arbitrarily set at 16 participants per cluster [9]. The choice of 16 individuals per cluster represents a compromise between data collection efficiency (larger number of participants per cluster) and reducing the DE (smaller number of participants per cluster). Piloting indicated that our research team could, on average, collect 16 cases in a day, but this would vary by time of year and the occurrence of diarrhea outbreaks.

### Zinc Treatment Scale Up

Under the title “Scaling Up Zinc for Young Children (SUZY) Project,” preparations and the eventual implementation of a national campaign in support of zinc treatment for childhood diarrhea was carried out over a period of 3 y (2003–2006). The campaign targeted children 6 mo to 5 y of age. This was a national effort, involving several public and private agencies, with coordination and financial support provided through the SUZY Project. There were no parallel campaigns occurring. Within the Bangladesh Ministry of Health and Family Welfare (MOHFW), a National Advisory Committee (NAC), chaired by the Health Secretary, was created. This committee was represented by the primary health care division of the MOHFW, the Bangladesh Pediatric Association, and country representatives from WHO and UNICEF. An implementation subcommittee was created to make specific recommendations to the NAC, including a zinc treatment policy statement, over-the-counter and advertising permits, and private sector participation. Private sector involvement included the selection of a pharmaceutical laboratory to produce and distribute a dispersible zinc tablet formulation, a marketing agency, and private provider groups, such as the Bangladesh Pediatric Association and the Bangladesh Village Doctors Association. Inclusion of these professional associations was critical given considerable doubt existed in the beginning about zinc safety and over-the-counter sales.

Following the selection of a pharmaceutical laboratory (ACME Pharmaceuticals, Ltd) a technology transfer from the French nutrition firm holding the patent for the dispersible zinc formulation (Nutriset Ltd) was arranged and eventually completed. Following the technology transfer, this included yearly scheduled quality control visits. ACME Pharmaceuticals then applied and obtained from the MOHFW Drug Administration for the following in the sequence listed: approval of the dispersible zinc tablet formulation, brand name (Baby Zinc) and packaging design, pricing (18 taka or approximately US$0.25 for a ten-tablet blister pack), an over-the-counter waiver, and a permit to advertise nationally on TV and radio.

Prior to initiating the national scale up several gaps in knowledge were identified and protocols prepared to provide the needed information. These included: (1) a phase IV safety and side effects studies carried out among children attending the ICDDR,B Dhaka hospital [10],[11]; (2) formative studies to better understand household diarrhea management decisions, treatment recommendations made by providers, the influence of drug salesmen, and knowledge of zinc and other micronutrients; (3) the acceptability of the dispersible tablets and adherence to preparation instructions [12]; and (4) a national survey in rural, urban slum, and nonslum populations to determine current childhood diarrhea management practices, health seeking behaviours, and expenditures [13]. These studies enabled us to reassure stakeholders that zinc treatment is safe, but associated with a low risk of nausea and vomiting. The results from the formative studies guided the preparation of messages for caregivers and providers. On the basis of these interviews and focus group discussions we created a frequently asked questions data bank and a uniform set of responses (available at www.icddrb.org). From the surveys we learned that over 90% of parents sought help from private sector providers and over 70% of the time it was with an unregulated provider (village doctor or drug vendor). It was this information that led to the decision to focus the campaign on caretaker decision making and availability of the zinc tablets in the private sector. Nevertheless, all public sector district health and family welfare centres were provided zinc tablets free of charge.

Prior to and following the national launch of the scale-up campaign, training sessions in diarrhea management in line with the revised WHO/UNICEF guidelines were conducted. These were tailored for pediatricians, general MBSS physicians, medical schools, and unregulated providers. For the latter, a 30-min training video was prepared. ACME drug salesmen provided verbal information and distributed a specially prepared pamphlet to private providers.

Because the large majority of parents interviewed identified TV as their primary source of information, the marketing campaign focused on this medium, but also prepared messages for radio, newspapers, billboards, and buses. In rural settings zinc promotion additionally included the sponsoring of cultural events and courtyard meetings. Four 30-s TV advertisements were prepared, the first being a “teaser” and the other three informing listeners of Baby Zinc treatment of diarrhea. These advertisements were broadcasted on Bangladesh National Television, which reaches all parts of Bangladesh. The messages included in the mass media promotions included awareness of zinc treatment for childhood diarrhea and its sanctioning by health providers in Bangladesh. All promotional activities linked zinc treatment to the continued use of ORS. Furthermore, as ORS is stopped once the diarrhea subsides, an additional message to continue the zinc for a full 10 d was included. Children 6 mo to 5 y of age were targeted.

### Survey Interviews

Twelve trained field research assistants divided into two teams carried out the household interviews and completed a 36 to 40 item questionnaire, depending upon the survey round (Text S1). Specific household management practices were documented first, followed by knowledge questions, including the question “Prior to this illness were you aware that zinc can be used to treat diarrhea in your child?” The recommended zinc treatment is 20 mg per day for 10 d, either as a dispersible tablet or 5-cc syrup formulation. In addition to asking whether zinc was used, we asked for how many days. Interviewers carried a laminated chart with photos of all syrup and tablet formulations being sold in Bangladesh to assist interviewees to identify the product used, In households where the purchased blister pack or bottle was available, we directly visualized them to confirm the reported number of days of use. Credit was given for any zinc formulation given.

Each of the ten selected survey sites required 7–10 d to complete and each round therefore required 3–4 mo. Timing was affected by seasonal floods, however the surveys remained on schedule for the 23 mo of follow-up monitoring. The interviews addressed the diarrhea illness history, health seeking behaviours, home management practices, illness-related expenditures, and sociodemographic characteristics of the households.

### Analysis

Data were entered and verified using SPSS-PC version 12. These files were then converted to STATA-PC version 10.0 for all analyses. Data files were checked for outliers and reduced categorical variables were generated. The analyses were stratified by location of residence into urban (Dhaka city corporation slum and nonslum districts), municipal, and rural households. To assess differences in categorical outcomes crude relative risks, 95% confidence intervals, and chi-square statistical comparisons of proportions were calculated. Of particular interest was the identification of disparities in the use of zinc by gender, geographic location, and income status of the household. Income status was estimated by determination of a household asset score based upon ownership of consumer items, dwelling characteristics, toilet facilities used, and other household characteristics that are related to wealth status [14],[15]. Each asset is assigned a weight generated through principal components analysis and then standardized scores assigned. The hypotheses we tested were that significant (*p*<0.05) differences in the likelihood of receiving zinc treatment for childhood diarrhea would be found favouring males, higher income, and urban nonslum households. Disparities were tested for significance applying Pearson chi-square statistics. To asses the magnitude and trends in income disparities, concentration index curves were determined for each follow-up period [16].

## Results

Seven survey rounds were completed with the range in the 2-wk prevalence (*p*) of diarrhea and the number of cases (*n*) surveyed by location as follows; urban slums *p* = 0.20–0.25 and *n* = 642–646; urban nonslums *p* = 0.17–0.23 and *n* = 641–658; municipalities *p* = 0.15–0.19 and *n* = 965–979; rural *p* = 0.19–0.23 and *n* = 962–976. The mean age of the children at each survey ranged from 26.3±0.3 mo to 27.4±0.3 mo and the percentage of cases being female ranged from 44.2% to 46.6%. Of all cases identified, 98.5% were enrolled in the surveys.

**Figure 2:**
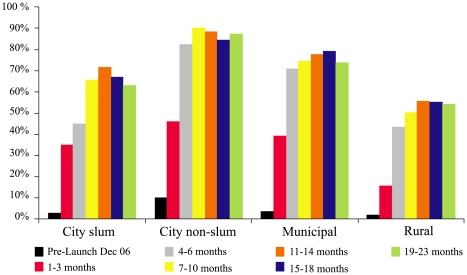
Caretaker awareness of zinc treatment for childhood diarrhea before and up to 23 mo following the onset of the mass media campaign.

Figure 2 summarizes caretaker awareness of zinc as a treatment for childhood diarrhea over time stratified by the location of the household. At baseline, prior to the launch of the mass media campaign, but following multiple workshops with licensed pediatricians, awareness was under 5% for all but urban, nonslum caregivers (99% mothers). In all locations awareness rapidly increased following the onset of mass media zinc promotion, reaching peak levels by 10 mo into the campaign. Among the urban slum and rural populations surveyed, zinc treatment awareness reached 65% and 55%, respectively, by 23 mo.

**Figure 3:**
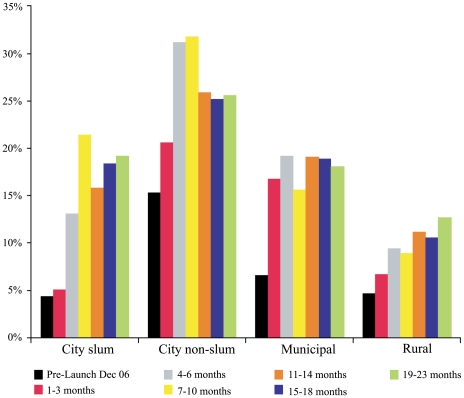
The proportion of children receiving zinc treatment by location of household before and up to 23 mo following the onset of the mass media campaign.

As illustrated in Figure 3, the actual use of zinc falls far short of awareness. With the exception of urban nonslum children, few received zinc prior to the national mass media launch. By the second year of the national scale-up campaign approximately 25% to 30% of urban nonslum, 15% to 20% of urban slum or municipal, and 9% to 13% of rural children were receiving zinc for their diarrheal illness episode. In urban nonslum and municipal households the use of zinc levelled off by the end of the first year, while a steady increase in zinc coverage has been observed in rural and urban slum areas. Table 1 summarizes the proportion of children who received zinc stratified by the type of provider seen during the final, 19- to 23-mo survey round; this is further subgrouped by whether a tablet or syrup formulation was received. Children seen by a private, MBBS provider were the most likely to receive zinc, at nearly 40%. Dispersible zinc tablets accounted for 60% of all zinc purchased.

**Table 1:** The proportion of children receiving zinc treatment and among these children the formulation taken by type of provider seen at 19–23 mo post-launch of the scale-up campaign.

Provider	Child		Formulation Taken	
	*n* Children Seen	Received Zinc (%)	Tablet (%)	Syrup (%)
Private, unlicensed village doctors or drug vendors	1,670	18.1	73.5	26.5
Private, licensed MBBS doctors	372	39.2	43.2	56.8
Public sector, MOHFW health providers	220	25.9	49.1	50.9
Overall	2,262	20.3	60.9	39.1

72% of the children surveyed were seen by a provider for their diarrheal illness.

**Figure 4:**
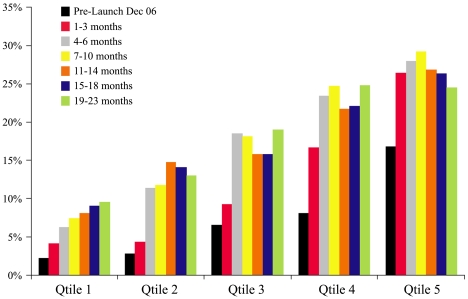
Changes in zinc coverage over time by household wealth asset quintile (1 lowest, 5 highest).

**Figure 5:**
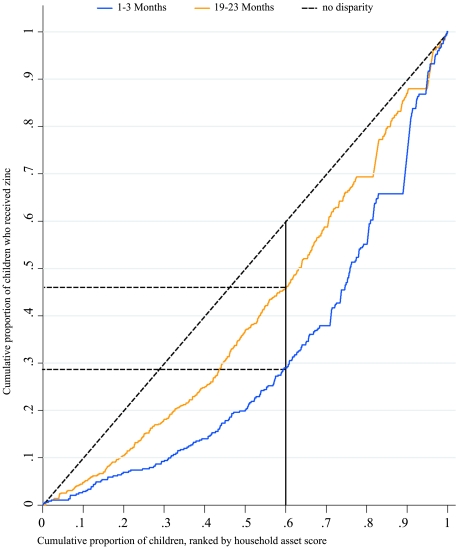
Income disparities in receiving zinc treatment as measured by concentration index curves 1–3 mo and 19–23 mo following the onset of the mass media campaign. Distribution of use of zinc is equal among income levels if the concentration curve coincides with diagonal. During the 1–3 month interval the 60% of poorest households accounted for 28% of the zinc treatments received, while from 19–23 mo they accounted for 46%.

In each survey period significant differences (*p*<0.001) in the use of zinc were observed favouring higher quintile wealth asset households (Figure 4). At 18 mo significant disparities in the likelihood of receiving zinc treatment on the basis of gender were limited to municipal households and favoured males (21% versus 16%, *p* = 0.024). No gender bias at any time interval was observed in urban slum and rural poor households. As can be seen from the concentration index curves summarized in Figure 5, income disparities in the use of zinc decreased over time. Referring to the figure, if there were no disparity in the use of zinc on the basis of household income status (asset score) then the poorest 60% of children would have accounted for 60% of the total zinc treatments received. At the outset of the mass media campaign (1–3 mo) this lower 60% accounted for only 28% of the zinc treatments received, but by the end of the second year (19–23 mo) this had risen to 46%.

**Figure 6:**
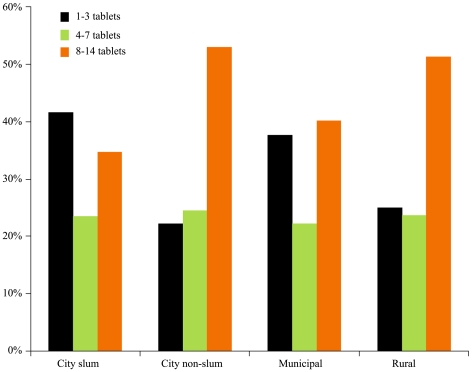
Number of zinc tablets purchased by caregivers during the final survey interval 19–23 mo following the mass media launch.

The cross-sectional design of these surveys does not permit a determination of the number of days the children received zinc treatment. As a proxy for duration of treatment, caretakers were asked the number of zinc tablets they purchased (Figure 6). By 19 to 23 mo following the launch of the mass media campaign in each of the population strata nearly 50% or more of zinc tablet purchases were for <8 d.

**Figure 7:**
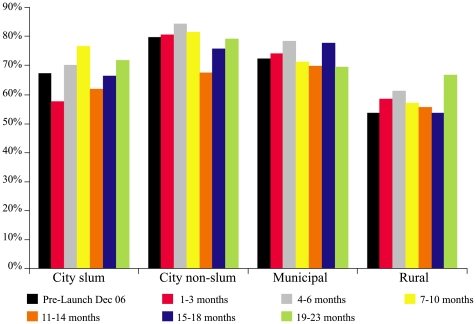
ORS utilization prior to and over the first 23 mo of the mass media zinc scale-up campaign.

Figure 7 summarizes changes in the use of ORS during the zinc scale-up campaign by household location. Variations in the estimated proportion of children receiving ORS occurred within each of the population strata over time, but no significant trend, upwards or downwards, in the use of ORS was observed. There was no significant change documented in the use of antidiarrheals. A significant reduction over the 2 y of follow-up in the use of antibiotics from 34.7% to 27.6% (*p*<0.001),was observed among urban nonslum households, but not in the other populations surveyed.

## Discussion

This 2-y follow-up monitoring of the national campaign to scale up zinc treatment of childhood diarrhea in Bangladesh has resulted in several observations of relevance to future scale-up efforts. This campaign largely focused on the promotion of zinc treatment among private sector providers, but with the strong support of the public health sector. The TV and radio promotion of zinc treatment through an electronic mass media campaign was able to rapidly attain high levels of awareness throughout the country. Progress in the actual use of zinc has been slower, with the early adoption primarily observed among urban, higher income households. Among rural and urban slum households, whose children stand to benefit the most from zinc, zinc treatment coverage steadily increased over time and the magnitude of disparity based upon income status was observed to have been reduced. Importantly, the scale-up campaign has not negatively impacted the use of ORS. An important, unmet challenge has been the failure to adhere to a 10-d course of treatment as evidenced by the fact over 50% of caregivers were sold seven or fewer days of zinc treatment.

A potential source of bias and limitation of this study is the populations surveyed, which may not be representative of hard to reach, more remote sites in Bangladesh. These sites were chosen because ICDDR,B researchers were known in these communities, support structures were in place, and local approval to conduct the surveys could be more rapidly obtained. The purpose of these surveys was to document trends in the use of zinc and changes in other practices within the stratified populations described. The findings may not, with confidence, be extrapolated to accurately estimate zinc coverage in all districts of Bangladesh. The sites chosen within each population strata are, nevertheless, typical Bangladeshi communities and we are confident that the observed trends in zinc coverage and reductions in disparities are indicative of what is occurring in Bangladesh as a whole. Bias in estimates of zinc treatment awareness may also have been introduced by repeatedly surveying in the same subdistricts. Those caretakers interviewed may have discussed the experience with relatives or neighbours, including the mention of zinc treatment. Households with a repeated case of diarrhea were not replaced because it was concluded their exclusion would lead to a biased selection of healthier children in subsequent surveys. The sites surveyed contain an estimated population of nearly 1.5 million children under 5 y of age. This was felt to be a large enough population base to minimize biased estimates of zinc awareness and would not have affected zinc coverage estimates. A strength of the surveys was the selection of households where a child had an active or recent diarrhea episode of at least 2-d duration. This eliminated transient, less important episodes and responses were based upon actual practices.

Promotion among health providers followed two strategies: half-day diarrhea training workshops and product promotion by drug salesmen. There are estimated to be over 200,000 health providers in Bangladesh, thus the challenge of reaching them all through workshops is not realistic. We therefore placed an early emphasis on sensitizing and training recognized leaders, such as pediatricians and educators. Less well-trained providers tend to look up to pediatricians and copy their practices. Among the unlicensed providers a training of trainers approach was used. While this set of surveys cannot document the proportion reached, it is likely the majority of health providers remain poorly informed about zinc treatment. Reliance on drug salesmen also has limitations. These individuals and the systems within which they work are profit oriented and based upon prescription medications. Zinc is cheap and it is an over-the-counter product. Not surprisingly, drug salesmen will be more inclined to promote higher priced products. Promotion and distribution of zinc through alternative systems, for example bottled water distribution networks, would reach a far greater number of outlets and increase its availability within rural or urban communities,

Rogers' diffusion of innovation theory is useful for understanding progress with scaling up health interventions in the general population over time. The theory describes the adoption of new innovations as passing through five stages of decision making—awareness, interest, evaluation, trial, and adoption [17]. At any stage a consumer, in our case providers or caretakers, can choose to reject the innovation. The mass media campaign was able to achieve high levels of awareness and probably interest among all segments of the Bangladeshi population. Where it has fallen short is in the transition from awareness to practice (trial and adoption). This gap highlights an important limitation of electronic media, which does not benefit from interpersonal communication, thus showing the need to link mass media messages with personal messages coming from health providers or other influential members of a community. The content of the initial commercials aired in this campaign repeatedly focused on awareness and health provider sanctioning of zinc treatment and not on household decision making. Towards the end of the second year of the campaign the media messages were altered to encourage household level decision making and enhancing self-efficacy to try zinc [18]. This strategy and interpersonal communication with early adopters of zinc treatment are expected to further increase coverage. The choice of electronic media in this scale-up campaign was, in part, based on its on its previous success in promoting the use of oral rehydration therapies (ORT) [19]. Nonetheless, ORTs and zinc share several characteristics that make them amenable to scaling up through mass media promotion. Both are fairly simple interventions that are easily learned and applied in the home. They are also relatively inexpensive and within the range of typical household diarrheal illness expenditures among Bangladeshi households [13].

Early adopters of new innovations are known to be better educated, of higher income status, and have greater access to mass media [17],[20]. We were able to monitor adoption of zinc treatment by household wealth asset quintiles. Throughout the 2 y of follow up children from higher wealth asset households were more likely to receive zinc treatment. At the outset of the campaign children in the highest quintile, when compared to the lowest quintile households, were seven times more likely to receive zinc. At the end of the second year this disparity had been reduced to less than three times as likely. This reduction in income disparity is further illustrated by the change in the concentration index curves from the beginning to the end of the follow-up monitoring.

Given the preventive effects of zinc are likely to require 8–10 d of treatment, the observation that over half of the children are receiving less than the required amount remains an important, unmet challenge. The mass media messages did include a parent directed reminder to give zinc for 10 d. Unfortunately, in Bangladesh drug vendors commonly sell antibiotics and other curative medications to cover only a few days. If a child remains ill, they return to purchase additional, often alternative, medication. Parents have little or no experience with continuing medications once their child appears to be cured. This behavioural change challenge currently lacks adequate scientific guidance.

All mass media messages in this campaign linked zinc to the use of oral saline. This connection is important, because zinc is an adjunct to and not a replacement for oral saline or other approved rehydration therapies. Future studies need to clarify whether linking zinc to oral saline may lead to caregiver misunderstanding, given they are instructed to discontinue the latter once the diarrhea subsides. Adherence would also be improved if it could be demonstrated that shorter duration zinc treatment schedules have equivalent clinical efficacies.

In summary, the national scale up of zinc treatment for childhood diarrhea in Bangladesh, the first national campaign to be undertaken, has met with modest success over the initial 2 y of promotion and has not had a detrimental effect on the use of ORT. While the relative benefits of provider versus caretaker mass media–focused promotion cannot be separated in this study, it is concluded that both strategies are important to undertake. To improve upon the overall coverage attained of about 20% of childhood diarrhea episodes will require greater, more consistent support from health providers and strengthened demand for zinc treatment from caregivers. Both of these objectives would benefit from an improved understanding of health system constraints as well as societal, household, and provider characteristics facilitating sustained behavioural change leading to the successful scale up of zinc and other life-saving interventions.

## Supporting Information

Text S1 Survey questionnaire.
(0.17 MB DOC)

Text S2 Study protocol.
(0.33 MB DOC)

## Abbreviations

MOHFW: Bangladesh Ministry of Health and Family Welfare
ORS: oral rehydration salt
ORT: oral rehydration therapies
