# Supplementary material for: Impact Monitoring of the National Scale Up of Zinc Treatment for Childhood Diarrhea in Bangladesh: Repeat Ecologic Surveys
Source: PLoS Med. 2009 Nov 3;6(11):e1000175. doi: 10.1371/journal.pmed.1000175 (PMC2765636; doi:10.1371/journal.pmed.1000175)
Supplement: Text S1 — Survey questionnaire. (0.17 MB DOC) [file pmed.1000175.s001.doc]

ICDDR,B: Centre for Health and Population Research Monitoring the Impact of the SUZY Project Role-out

**of Zinc as a Treatment for Childhood Diarrhea**

**National Coverage (Cluster) Survey**

Case ID

Date of interview:____dd/ ____mm______yy Round # (Circle) 1 2 3

Interviewer ID ___________

1. Site ID: Dhaka 05. Khulna

01. Zone 1 (Kamalapur) 06. Sylhet

02. Zone 8 (Mirpur) 07. Comilla

03. Zone 3 (Lalbagh) 08. Mirsarai (Rural)

04. Zone 5 (Green Road) 09. Abhoynagar (Rural)

10. Hobiganj (Rural)

Cluster # ___ ___

1. Child’s ID: ___ ___

**Demographic Information:**

1. Age of the child: _____ months
2. Child’s gender: 1. Male 2. Female
3. Father’s occupation:

01.Unemployed 02. Day labor 03. Rick/Van puller 04. Fisherman

05. Boatman 06. Carpenter 07. Painter 08. Farmer 09. Driver

10. Petty Business 11. Teacher 12. Service holder

13. Business/Contractor 14. Mason 15. Garment Worker 16. electrician

17. aborad 77. Others

88. Died, sepa. Div 99. Don’t know/missing

1. Father’s education (years completed):_____________
2. Mother’s age:________ years
3. Mother’s occupation:

01. House wife 02. Day labor 03. Teacher 04. Service holder

05. Handicrafts 06. Garment worker 07. Maid servant 08. Tailor

09. small business 77. Others 88. Died, Div. Sepa 99. Don’t know/Missing

1. Mother’s education (completed years):________
2. No. of living children under 10 years old

of this mother (excluded the interviewed child):________

**Socio-Economic Information:**

1. Construction of the dwelling house:

Mud/Thatch=0, Bamboo=1, Wood=2, Tin=3, Cement=4

Tally=5, Others=7

12a. Floor: _______ 12b. Wall: ________ 12c. Roof: ________

1. Number of rooms in the dwelling house:____ ____

1. Does your family have?: 1. yes 2. no

- - - 1. Radio ___

- - - 1. TV ___
      2. Electricity ___

- - - 1. Bicycle ___
      2. Sewing machine ___

- - - 1. Motorcycle ___
      2. Almirah ___
      3. Table ______
      4. Chair/Bench ____

10. Watch/Clock ____

11. khat ______

12. Chaki ________

13. Telephone/mobile ______

1. What type of latrine does your family have?

1. No facility/Bush/Field 2. Hanging latrine

3. Pit latrine 4. Water sealed/Slab latrine

5. Septic tank/Modern latrine 7. others

1. In an average week how much does your family spend on food?

_________________ Taka.

1. Prior to this illness, were you breastfeeding your child?

1. yes 2. no

17a. If yes, are you continuing to breast feed?

1. yes 2. no

**History of current/recent diarrhea illness of your child**

1. What type of diarrhea does/did your child have? 1. Yes 2. No
   - - 1. Watery
       2. Loose
       3. Mucoid
       4. Bloody
2. Is diarrhea continuing today with your child? 1. Yes 2. No
3. How many days has your child had suffered from diarrhea? ____
4. Did you seek help from a health care provider for

your child’s illness? 1. Yes 2. No If no, go to Q. 24

Provider’s consultation fee

1. If Q. No. 21 is yes then type of provider seen for this illness

1. MBBS 1. yes 2. no

2. MOHFW worker 1. yes 2. no

3. NGO Worker 1. yes 2. no

4. Unlicensed allopath 1. yes 2. no

5. Homeopath 1. yes 2. no

6. Drug vendor 1. yes 2. no

7. Traditional healer 1. yes 2. no

8. Other ___________________ 1. yes 2. no

N/A = 8 Dont Know = 9

22a. If visit more than one provider then whom was first?

1. Sector of provider consulted and cost (user fee) for the consultation?

1. yes 2. no 9. Dont know 8. N/A

- - - 1. Government
      2. NGO
      3. Private: Licensed
      4. Unlicensed

**ORS Treatment:**

1. Was ORS given to your child? 1. yes 2. no 9. Don’t know
   1. if yes, for _________days

- 1. if yes, how much did you spend on ORS? __________Taka ,

Don’t know = 999 NA = 888

1. Other than ORS, did you give any other rehydration solution

to your child? 1. yes 2. no Dont know = 9 NA = 8

If yes:

(1). Home made ORT:

(2). SSS

(3). Water

(4) fruit juice/suger water/coconut water

(5).Flattened rice water/rice water __________________

(6) Other

25a. Did you continue to feed (normal) your child throughout

the diarrheal illness (other than rehydrationfluids)?

1. yes 2. no

# Zinc Treatment:

1. Was zinc given to your child? 1. yes 2. no (go to Q.31) 9.Dont know(go to Q.31)

26a. if yes, for how many days? ____ days NA = 8

26b if not given 10 days, why?

27. Was the zinc a 1. tablet or 2. syrup? NA = 8

27a. If syrup, how many tea spoon/cc is given daily?

27b. If tablet, how many tablets were you sold?

28 Did your child vomit within 30 minutes following zinc treatment?

1. yes 2. no NA=8

28a If yes, have you stopped zinc treatment due to vomiting?

1. yes 2. no NA=8

1. What was the zinc product’s name (or show me the strip/bottle)?

Name of zinc: _______________ don’t know = 99 NA = 88

1. How much did it cost you? __________Taka,

don’t know = 999 NA = 888

30a. At this price, would you buy zinc again the next time your child  has diarrhea? 1. yes 2. no 9. not sure 8. NA

1. From where was it obtained? 1. Hospital 2. Clinic 3. Private provider 4. Drug vendor/Pharmacy 5. Other __________

8. NA

**Antibiotic Treatment** (Show picture chart)

32. Was an antibiotic given to your child? 1. Yes 2. No 3. don’t know

Go to 35

33. If yes, name of antibiotic ________________________________

34. How much did it cost you? __________ Taka,

Don’t Know = 99 NA = 88

35. In addition to Zinc, ORS or antibiotics, please tell me the name or show me other medicines given for this illness and the cost of this medicine.

None skip to Q. 36

35a. Name of medicine Cost (Taka)

__________________ ________________

__________________ ________________

__________________ ________________

Total cost _________ Taka

36. Was your child referred to a hospital or clinic for this illness?

1. Yes, hospital ___ 2. Yes, clinic ___ 3. No Dont know

stop interview

1. How much it cost you? _____________Taka

Don’t know = 9999

NA = 8888

38. Interviewer’s assessment about socio economic status of the household

- - - 1. very poor
      2. poor
      3. medium
      4. well off

**Thank you**
