# Supplementary material for: Impact Monitoring of the National Scale Up of Zinc Treatment for Childhood Diarrhea in Bangladesh: Repeat Ecologic Surveys
Source: PLoS Med. 2009 Nov 3;6(11):e1000175. doi: 10.1371/journal.pmed.1000175 (PMC2765636; doi:10.1371/journal.pmed.1000175)
Supplement: Text S2 — Study protocol. (0.33 MB DOC) [file pmed.1000175.s002.doc]

| **Centre for Health & Population Research** | | | | RRC APPLICATION FORM | | |
| --- | --- | --- | --- | --- | --- | --- |
| RESEARCH PROTOCOL **NUMBER:** | **FOR OFFICE USE ONLY** | | | | | |
|  | RRC Approval: | | | Yes / | No | Date: |
|  | ERC Approval: | | | Yes / | No | Date: |
|  | AEEC Approval: | | | Yes / | No | Date: |
| **Protocol Title:** Monitoring the Impact of the SUZY Project Role-out of Zinc  **as a Treatment for Childhood Diarrhea** | | | | | | |
| **Short title (in 50 characters including space):** Impact of scaling up zinc for diarrhea treatment | | | | | | |
| **Theme: (Check all that apply)**  Nutrition  Emerging and Re-emerging Infectious Diseases  Population Dynamics  Reproductive Health  Vaccine Evaluation  HIV/AIDS | | | Environmental Health  Health Services  Child Health  Clinical Case Management  Social and Behavioural Sciences | | | |
| **Key words:** Scale up, zinc, childhood diarrhea, equity | | | | | | |
| **Relevance of the Protocol:**  This is the first national scale up of zinc. Impact monitoring of desired and undesired practices, as well as for equity, are essential | | | | | | |
| **Centre’s Priority** **(as per Strategic Plan, to be imported from the attached Excel Sheet):**  Accomplishment #1: Cost effective strategies for zinc therapy in children | | | | | | |
| **Programmes:**  Child Health Programme  Nutrition Programme  Programme on Infectious Diseases & Vaccine Science  Poverty and Health Programme | | | Health and Family Planning Systems Programme    Population Programme  Reproductive Health Programme  HIV/AIDS Programme | | | |
| **Principal Investigator (Should be a Centre’s staff)**  **Charles P. Larson**  **Address (including e-mail address):**  clarson@icddrb.org | | | **DIVISION:**  CSD  LSD  HSID  PHSD | | | |
| **Co-Principal Investigator(s): Internal** | | | | | | |
| **Co-Principal Investigator(s): External:**  (Please provide full official address including e-mail address and Gender) | | | | | | |
| **Co-Investigator(s): Internal:**  Unnati Rani Saha | | | | | | |
| **Co-Investigator(s): External**  (Please provide full official address including e-mail address and Gender) | | | | | | |
| **Student Investigator(s): Internal (Centre’s staff):** | | | | | | |
| **Student Investigator(s): External:**  (Please provide full address of educational institution and Gender) | | | | | | |
| **Collaborating Institute(s): NONE** | | | | | | |
| **Population: Inclusion of special groups (Check all that apply):**   | Gender  Male  Female  Age  0 – 4 years  5 – 9 years  10 – 19 years  20 – 64 years  65 + | Pregnant Women  Fetuses  Prisoners  Destitutes  Service Providers  Cognitively Impaired  CSW  Others (Parents)  Animal | | --- | --- |   NOTE It is the policy of the Centre to include men, women, and children in all research projects involving human subjects unless a clear and compelling rationale and justification (e.g. gender specific or inappropriate with respect to the purpose of the research) is there. Justification should be provided in the `Sample Size’ section of the protocol in case inclusiveness of study participants is not proposed in the study. | | | | | | |
| **Project/study Site (Check all the apply):**  Dhaka Hospital  Matlab Hospital  Matlab DSS Area  Matlab non-DSS Area  Mirzapur  Dhaka Community  Chakaria  Abhoynagar | | Mirsarai  Patyia  Other areas in Bangladesh Mirpur, Kamalapur, Sylhet  Outside Bangladesh  Name of Country:  Multi Centre Trial  (Name other countries involved): | | | | |
| Type of Study (Check all that apply):   | Case Control Study  Community-based Trial/Intervention  Program Project (Umbrella)  Secondary Data Analysis  Clinical Trial (Hospital/Clinic)  Family Follow-up Study | Cross Sectional Survey  Longitudinal Study (cohort or follow-up)  Record Review  Prophylactic Trial  Surveillance/Monitoring  Others: | | --- | --- |   NOTE: Does the study meet the definition of clinical studies/trials given by the International Committee of Medical Journal Editors (ICMJE)? Yes  No    Please note that the ICMJE defined clinical trial as “*Any research project that prospectively assigns human subjects to intervention and comparison groups to study the cause-and-effect relationship between a medical intervention and a health outcome*”.    If YES, after approval of the ERC, the PI should complete and send the relevant form to provide required information about the research protocol to the Committee Coordination Secretariat for registration of the study into websites, preferably at the [www.clinicaltrials.gov](http://www.clinicaltrials.gov/). It may please be noted that the PI would require to provide subsequent updates of the research protocol for updating protocol information in the website. | | | | | | |
| **Targeted Population (Check all that apply):**  No ethnic selection (Bangladeshi)  Bangalee  Tribal group | | Expatriates  Immigrants  Refugee | | | | |
| **Consent Process (Check all that apply):**  Written  Oral  None | | Bengali Language  English Language | | | | |
| **Proposed Sample Size:**  Sub-group (Name of subgroup (e.g. Men, Women) and Number   | Name | Number | Name | Number | | --- | --- | --- | --- | | (1) Rural | 960 | (3) City corporations slums | 640 | | (2) Urban municipalities | 960 | (4) City corporation non-slum | 640 |   Total sample size: 3200 | | | | | | |
| **Determination of Risk: Does the Research Involve (**Check all that apply**):**   | Human exposure to radioactive agents?  Fetal tissue or abortus?  Investigational new device?  (specify:      )  Existing data available from Co-investigator | Human exposure to infectious agents?  Investigational new drug  Existing data available via public archives/sources  Pathological or diagnostic clinical specimen only  Observation of public behaviour  New treatment regime | | --- | --- | | | | | | | |
| **Could the information recorded about the individual if it became known outside of the research:**   | Yes | No | Is the information recorded in such a manner that study participants can be identified from information provided directly or through identifiers linked to the study participants? | | --- | --- | --- | | Yes | No | Does the research deal with sensitive aspects of the study participants’ behaviour; sexual behaviour, alcohol use or illegal conduct such as drug use? |  | Yes | No | Place the study participants at risk of criminal or civil liability? | | --- | --- | --- | | Yes | No | Damage the study participants’ financial standing, reputation or employability, social rejection, lead to stigma, divorce etc.? | | | | | | | |
| **Do you consider this research (Check one):**  Greater than minimal risk  No more than minimal risk  Only part of the diagnostic test  Minimal Risk is "a risk where the probability and magnitude of harm or discomfort anticipated in the proposed research are not greater in and of themselves than those ordinarily encountered in daily life or during the performance of routine physical, psychological examinations or tests. For example, risk of drawing a small amount of blood from a healthy individual for research purposes is no greater than the risk of doing so as a part of routine physical examination". | | | | | | |
| **Yes/ No**  Is the proposal funded?  If yes, sponsor Name: (1) Bill and Melinda Gates Foundation    (2) | | | | | | |
| **Yes/No**  Is the proposal being submitted for funding?  If yes, name of funding agency: (1)  (2) | | | | | | |
| Do any of the participating investigators and/or member(s) of their immediate families have an equity relationship (e.g. stockholder) with the sponsor of the project or manufacturer and/or owner of the test product or device to be studied or serve as a consultant to any of the above? No  ***IF YES, a written statement of disclosure to be submitted to the Centre’s Executive Director.***  Dates of Proposed Period of Support Cost Required for the Budget Period ($)  (Day, Month, Year - DD/MM/YY)   | **Years** | **Direct Cost** | **Indirect Cost** | **Total Cost** | | --- | --- | --- | --- | | **Year-1** | 157,896 | 50,527 | 208,4230 | | **Year-2** |  |  | 0 | | **Year-3** |  |  | 0 | | **Year-4** |  |  | 0 | | **Year-5** |  |  | 0 | | **Total** | 0 | 0 | 0 |     Beginning Date : 01/09/06    End Date : 15/06/07 | | | | | | |
| **Certification by the Principal Investigator**  I certify that the statements herein are true, complete and accurate to the best of my knowledge. I am aware that any false, fictitious, or fraudulent statements or claims may subject me to criminal, civil, or administrative penalties. I agree to accept the responsibility for the scientific conduct of the project and to provide the required progress reports including updating protocol information in the SUCHONA (Form # 2) if a grant is awarded as a result of this application.  ___________ ____________  **Signature of PI Date** | | | | | | |
| **Approval of the Project by the Division Director of the Applicant**  The above-mentioned project has been discussed and reviewed at the Division level as well by the external reviewers. The protocol has been revised according to the reviewers’ comments and is approved.   | Charles P. Larson |  | 5 July, 2006 | | --- | --- | --- | | Name of the Division Director | Signature | Date of Approval | | | | | | | |

Table of Contents

[RRC APPLICATION FORM 1](#__RefHeading___Toc132346844)

[Project Summary 6](#__RefHeading___Toc132346845)

[Description of the Research Project 7](#__RefHeading___Toc132346846)

[Hypothesis to be Tested: 7](#__RefHeading___Toc132346847)

[Specific Aims: 7](#__RefHeading___Toc132346848)

[Background of the Project including Preliminary Observations 7](#__RefHeading___Toc132346849)

[Research Design and Methods 9](#__RefHeading___Toc132346850)

[Sample Size Calculation and Outcome Variable(s) 11](#__RefHeading___Toc132346851)

[Facilities Available 12](#__RefHeading___Toc132346852)

[Data Safety Monitoring Plan (DSMP) 12](#__RefHeading___Toc132346853)

[Data Analysis 12](#__RefHeading___Toc132346854)

[Ethical Assurance for Protection of Human Rights 13](#__RefHeading___Toc132346855)

[Use of Animals 13](#__RefHeading___Toc132346856)

[Literature Cited 13](#__RefHeading___Toc132346857)

[Dissemination and Use of Findings 14](#__RefHeading___Toc132346858)

[Collaborative Arrangements 14](#__RefHeading___Toc132346859)

[Biography of the Investigators 15](#__RefHeading___Toc132346860)

[Biography of the Investigators 17](#__RefHeading___Toc132346861)

[Detailed Budget 18](#__RefHeading___Toc132346863)

[Budget Justifications 18](#__RefHeading___Toc132346864)

[Other Support 18](#__RefHeading___Toc132346865)

[Appendix 1: Voluntary Consent Form 19](#__RefHeading___Toc132346866)

[Appendix 2: Comments of External Reviewers 20](#__RefHeading___Toc132346867)

[Appendix 3: Response to External Reviewer’s Comments](#__RefHeading___Toc132346868)

[Appendix 4: Abstract Summary covering eight points specified by the ERC](#__RefHeading___Toc132346869)

Appendix 5: Detailed Budget

Appendix 6: Questionnaire

[Check-List](#__RefHeading___Toc132346870)

Check here if appendix is included

| Project Summary Describe in concise terms, the hypothesis, objectives, and the relevant background of the project. Also describe concisely the experimental design and research methods for achieving the objectives. This description will serve as a succinct and precise and accurate description of the proposed research is required. This summary must be understandable and interpretable when removed from the main application. **(Please keep as brief as possible).** |
| --- |
| Principal Investigator(s): Charles Larson |
| Research Protocol Title: Monitoring the Impact of the SUZY Project role-out of Zinc as a Treatment for Childhood Diarrhea |
| Total Budget US$: 208,423 Beginning Date : 01/09/06 Ending Date: 15/06/07 |
| This is a repeat, ecologic survey to document changes in zinc coverage for childhood diarrhea, coinciding with the national roll-out of “Baby Zinc”. The surveys have been stratified by location; rural, urban municipalities, and city coprportation slum and non-slum populations.  In addition to zinc coverage, the surveys will monitor changes in the use of ORS or antibiotics, expenditures on diarrheal illnesses and other management practices. The surveys have been powered to identify disparities in care based upon gender, income and location of households.  The survey field sites have been purposively selected in order to facilitate comparisons with the baseline national coverage survey carried out in 2003/4. These include rural upazilla (Mirsarai, Abhoynagar, Hobiganj), urban municipalities (Khulna, Sylhet, Camila), and City Corporation zones (slum: kamalapur, Mirpur and non-slum Green--------- The same interviewer administered questionnaire will be used.  The results of this study will provide guidance for public, private and NGO delivery systems as they jointly endeavor to reach all under-five children with zinc treatment of any diarrheal disease episode. |
| KEY PERSONNEL (List names of all investigators including PI and their respective specialties)     | Name | Professional Discipline/ Specialty | Role in the Project | | --- | --- | --- | | 1. Charles P. Larson | Health services research | PI | | 1. Unnati Rani Saha | Statistician | analyses, data management | | 1. Hazera Nazmun | Field studies | Coordination of survey conduct | |

# Description of the Research Project

##

## Hypothesis to be Tested:

Concisely list in order, the hypothesis to be tested and the Specific Aims of the proposed study. Provide the scientific basis of the hypothesis, critically examining the observations leading to the formulation of the hypothesis.

Over the planned 8 months of observation;

H1: That there will be a significant, upward trend in use of zinc for the treatment of childhood diarrhea.

H2: That there will be a significant downward trend in the use of antibiotics for the treatment of non-bloody childhood diarrhea

H3: That there will be disparities in zinc treatment coverage on the basis of household income status. gender oand location (rural, urban, city corporation slum and non-slum populations).

## Specific Aims:

Describe the specific aims of the proposed study. State the specific parameters, biological functions/ rates/ processes that will be assessed by specific methods.

Coinciding with the health care provider promotion and mass media marketing of "Baby Zinc" for the treatment of childhood diarrhea,

1. To monitor the proportion of under-five diarrheal episodes receiving zinc treatment and changes

over time.

2. To document changes in provider and caretaker practices. Included here are the use of ORS,

antibiotics, antidiarrheal and other prescribed treatments.

3. To monitor changes in household expenditures on diarrheal illnesses.

4. To monitor for disparities in zinc coverage by household income status, gender and location.

5. To assess impact in terms of need for/utilization of clinical services and hospitalizations.

## Background of the Project including Preliminary Observations

Describe the relevant background of the proposed study. Discuss the previous related works on the subject by citing specific references. Describe logically how the present hypothesis is supported by the relevant background observations including any preliminary results that may be available. Critically analyze available knowledge in the field of the proposed study and discuss the questions and gaps in the knowledge that need to be fulfilled to achieve the proposed goals. Provide scientific validity of the hypothesis on the basis of background information. If there is no sufficient information on the subject, indicate the need to develop new knowledge. Also include the significance and rationale of the proposed work by specifically discussing how these accomplishments will bring benefit to human health in relation to biomedical, social, and environmental perspectives.

Zinc deficiency has been found to be widespread among children in developing countries, occurring throughout Latin America, Africa, the Middle East and South Asia. It has been estimated that the annual Disability Adjusted Life Years (DALYs) lost due to zinc deficiency is over 57 million (1) . Children between 6 months to 5 years of age who receive zinc for the treatment of a diarrheal illness (20 mg/day for 10 days) recover faster, have a 30% reduction in the likelihood of developing prolonged diarrhea, and have an estimated 50% reduction in non-injury mortality over the next 6 months (2-8). In response to this evidence, WHO/UNICEF revised its childhood diarrhea management recommendations to include zinc in the treatment of any under-five child with a diarrheal illness (9). This evidence also sstimulated the develpoment of the Gates Foundation funded Scale Up of Zinc for Young Chuildren (SUZY) Project. The SUZY Project is now into its fourth and final year. The national roll-out of a zinc providedr promotion and mass media campaign in support of “baby zinc” is scheduled to begin in late September or early October, 2006.

As part of the Project monitoring plan a baseline national survey of childhood diarrhea management practices was carried out in 2004. Stratified by rural, urban and city corporation slum and non-slum populations, the aims of that survey were to document help seeking practices, treatments given, and illness expenditures. An important issue that the baseline survey and this follow-up survey addresses is that of equity. Most new health interventions are preferentially used by the more well-to-do, with the poor excluded and lacking access to new technologies. This project intends to monitor for the inequities (e.g. by gender and income) and provide guidance for pro-poor policy and programming decisions. Special efforts have been made to tailor media messages for the poor.

The baseline survey documented that throughout Bangladesh health seeking behaviors for childhood diarrhea are dominated by utilization of private sector providers (10). It is evident that unlicensed providers, whether “village doctors”, drug sellers or homeopaths, continue to be the preferred source of care, in particular among the rural poor. It was also found that significant variation in the type of provider seen occurs by where the child lived. In Bangladesh licensed providers are predominantly allopathic doctors (MBBS), while unlicensed providers are typically individuals with minimal or no formal training who imitate the prescribing patterns of trained, qualified doctors. Unlicensed providers (allopaths, drug sellers, homeopaths) were visited in over 90% of rural and over 75% of inner-city slum cases when care was sought. Unexpectedly, it was found that urban, non-slum when compared to poorer rural and slum households, were twice as likely to seek services from the public sector.

Disparities on the basis of income in care seeking behavior were identified in rural and urban populations. This was true for any provider as well as for a licensed allopath with significant trends (p<.001) favoring higher income households occurring throughout Bangladesh. When adjusted for host and illness characteristics, within urban households the most consistent predictors for seeing a licensed allopath were higher income, longer duration of illness and higher education of mothers. In rural households, where access to licensed providers is much more restricted, the most important predictors were younger age of child, longer duration of illness and mothers’ education. Gender disparites in provider utilization were not identified among rural households. When comparing female to male children, the crude estimates for seeking help from any provider or a licensed allopath favored males from city corporation, non-slum households. These disparities disappeared when the analysis was adjusted for other predictors of utilization. In contrast, within urban municipal households females were found to be 30% less likely to have been seen by a licensed allopath. No gender bias for provider utilization (licensed or unlicensed) was identified among the much poorer inner-city slum households.

With respect to total direct expenditures on a diarrheal illness, there was a consistent trend across all locations to spend more on male children, however this only reached statistical significance among city corporation, non-slum households. A similar trend was found for purchase of an antibiotic and may explain the expenditure disparities. Receipt of an antibiotic is closely correlated with having seen a provider and therefore these two outcomes will share similar predictors. This included higher income and maternal education, longer duration of illness, and younger age. An additional predictor of antibiotic use, as would be desired, was the finding that children with bloody diarrhea were twice as likely to receive an antibiotic.

The treatment protocol to be scaled up in Bangladesh is a 10 day course of 20 mg of zinc daily provided in either a dispersible tablet or syrup formulation. The tablets are converted from a solid tablet to a syrup with the addition of a few drops of water. The tablets will be packaged and sold in a 10 tablet blister pack. The choice of a 10 day treatment protocol is based upon expectations regarding the expected length of compliance (to be monitored) and current production capacities, which are set to produce 10 tablet blister packs.

The SUZY Project has already completed a great deal of provider promotion that has addressed zinc treatment of childhood diarrhea generically. Nearly all licensed and probably most unlicensed providers are aware of zinc as a treatment for childhood diarrhea and many are prescribing it. Thus, one purpose of this proposed follow-up survey ,in its first of three rounds, is to establish the post-provider promotion, but pre mass media coverage of zinc and to then monitor for change in diarrhea management practices as the Baby Zinc roll-out campaign is implemented. Equally important will be the monitoring of ORS and zinc use. Both outcomes will be stratified by income status, gender of child and location of residence.

## Research Design and Methods

Describe in detail the methods and procedures that will be used to accomplish the objectives and specific aims of the project. Discuss the alternative methods that are available and justify the use of the method proposed in the study. Justify the scientific validity of the methodological approach (biomedical, social, or environmental) as an investigation tool to achieve the specific aims. Discuss the limitations and difficulties of the proposed procedures and sufficiently justify the use of them. Discuss the ethical issues related to biomedical and social research for employing special procedures, such as invasive procedures in sick children, use of isotopes or any other hazardous materials, or social questionnaires relating to individual privacy. Point out safety procedures to be observed for protection of individuals during any situations or materials that may be injurious to human health. The methodology section should be sufficiently descriptive to allow the reviewers to make valid and unambiguous assessment of the project.

Study design: A repeat, ecologic survey in three rural upazillas, three municipalities and two city coprporation slum plus two non-slum zones is proposed. The survey team will complete three survey cycles per site, each cycle estimated to require 10 weeks to complete. The surveys will be carried out from September, 2006 through April, 2007 (SUZY Project funding ends in June, 2007).

Survey Populations

a. Rural: The sub-districts of Mirsarai, Abhoynagar and Hobiganj have been purposively selected. Within

each sub-district we will randomly select 20 clusters based upon a listing of all wards in the subdistrict.

All wards will be enumerated and then randomly selected.

b. Urban: The district municipalities of Khulna, Camila and Sylhet have been purposively selected. Twenty

clusters will be randomly selected within each municipality. Clusters are defined as geographic units

(“strata”) within the municipality that will be enumerated.

c. Dhaka City Corporation: slum zones of Kamalapur and Mirpur, non-slum zones (and wards) of Green

Road and Old Dhaka. Within each zone 20 clusters will be selected. Clusters are geographically defined

population“units” based upon the most recent census

Within each cluster a household census survey will be completed for the identification of any under -five child who has had a diarrheal ilness in the past two weeks of at least 2 days duration. The aim is to identify 16 cases per cluster.

Within each site, random cluster selection will be repeated at the time of each new survey round. The sampling framework is summarized in figure 1.

CHILDREN UNDER FIVE YEARS OF AGE

Diarrheal Disease Episode in past 2 weeks and

at least 48 hrs duration

Dhaka Urban Municipalities Rural Upazillas

2 slum zones Khulna Abhoynagar

2 non-slum zones Camila Mirsarai

Sylhet Hobiganj

random random random

20 clusters 20 clusters 20 clusters

per zone per municipality per upazilla

at 16 casesper cluster

n=320 cases per site

N= 640 slum N=960 N=960

N= 640 non-slum urban municipal rural cases

per round per round per round

Figure 1. Summary of sampling frame for cluster surveys

## Sample Size Calculation and Outcome Variable(s)

For each survey round (weeks 1 to10, 12 to 21 , 23 to 32 weeks) we are determining estimates of zinc, ORS and antibiotic use (prevalence). With each new interval we will be sampling from the same sites, but will carry out a new random selection of clusters in which cases will be identified.

As depicted in figure 1, for each survey round the number of subjects by site and location is as follows:

Location Sites N/site N/location

City corporations Slums x 2 320 640

Non-slums x 2 320 640

Urban Municipalities x 3 320 960

Rural Upazillas x 3 320 960

Based upon WHO EPI cluster survey methods, the design effect is estimated to be 2.0 (11)

a. Prevalence of zinc treatment by site: Setting confidence at 0.95 and a minimal detectable error of a 5% for a prevalence estimate within a site, assuming the baseline overall prevalence of zinc coverage to be 10%:

(Z _alpha_ )^2^ (P)(1-P)

N/site = x 2 (cluster adjustment design effect)

d ^2^

N/site = 276 cases

b. Trends in use of ORS and antibiotics by site: Setting confidence at 0.95 and a minimal detectable error of 10% around a prevalence of 50% of cases being treated with ORS or an antibiotic;

N/site = 198

d. Comparison of two proportions (hypothesis testing):

To examine whether zinc coverage in urban or rural locations is significantly different among males vs females, setting the level of conficence at .95 (one-sided), power at .80, minimal detectable difference 10%, assuming an overall prevalence of 25% coverage by the third cycle

(Z _alpha_ + Z _beta_)^2^ (P)(P-1) x 2

N/gender/location = x 2 (cluster adjustment design effect)

d ^2^

N/location = 461

Given these estimates, we will have the power to detect smaller differences in the rural and municipality

locations. In the City Corporations sites the power is adequate if we combine slum and non-slum populations. Keeping them separate will allow for a minimal detectable difference of 12%.

Measurement: An interviewer completed questionnaire essentially the same as the baseline questionnaire (questions on expenditures have been modivied to provide greater clarity on commodity purchases vs. service cahrges). . The questionniare requires about 30 minutes to complete. Questions cover host, illness and socio-demographic characteristics followed by household diarrhea management practices and expenditures on the identified case. Socioeconomic status will be estimated by determination of a household asset score based upon ownership of consumer items, dwelling characteristics, toilet facilities used, and other household characteristics that are related to wealth status (12). Each asset is assigned a weight generated through principal components analysis and then standardized scores assigned (13). For any household all asset scores will be summed and then sub-grouped into quartiles.

Sixteen FRAs will be trained to carry out case finding and household interviews. They will be supervised by two FROs. The entire data collection team will be supported by an NOA level co-investigator who has had experience with the baseline surveys.

## Facilities Available

Describe the availability of physical facilities at the place where the study will be carried out. For clinical and laboratory-based studies, indicate the provision of hospital and other types of patient’s care facilities and adequate laboratory support. Point out the laboratory facilities and major equipment that will be required for the study. For field studies, describe the field area including its size, population, and means of communications.

This study will be supported by the SUZY Project management team and office facilities will be provided by the HSID division. Logistical support will be provided by the field site managers in Abhoynagar, Mirsarai, Kamalapur and Mirpur.

## Data Safety Monitoring Plan (DSMP)

All clinical investigations (biomedical and behavioural intervention research protocols) should include the Data and Safety Monitoring Plan (DSMP) to provide the overall framework for the research protocol’s data and safety monitoring. It is not necessary that the DSMP covers all possible aspects of each elements. When designing an appropriate DSMP, the following should be kept in mind.

1. All investigations require monitoring;
2. The benefits of the investigation should outweigh the risks;
3. The monitoring plan should commensurate with risk; and
4. Monitoring should be with the size and complexity of the investigation.

Safety monitoring is defined as any process during clinical trails that involves the review of accumulated outcome data for groups of patients to determine if any treatment procedure practised should be altered or not.

**Not applicable**

## Data Analysis

Describe plans for data analysis. Indicate whether data will be analyzed by the investigators themselves or by other professionals. Specify what statistical software packages will be used and if the study is blinded, when the code will be opened. For clinical trials, indicate if interim data analysis will be required to monitor further progress of the study.

The investigative team will carry out the data analysis. Data entry and general management will be done by the Data Resources and Surveillance Unit of the Division. Data will be entered and analyzed using SPSS version 12.0. Absolute counts, proportions and means with 95% confidence intervals were calculated. These and the regression analyses will be completed using STATA version 9, cluster survey program , that accounts for potential within cluster homogeneity. The analyses will stratified by location of residence into rural, municipal and city corporation households. The latter are further sub-grouped as predominantly slum or non-slum populations. For differences in categorical outcomes crude relative risks and 95% confidence intervals will determined. Multiple logistic regression models were tested for the prediction of health seeking behaviours and antibiotic, ORS or zinc treatments received. Of particular interest is the identification of disparities in health provider utilization, diarrhea treatment practices and illness expenditures by gender, asset quartile (household wealth) and geographic location of the household (rural, municipal or city corporation). The hypotheses to be tested are that significant (p<.05) differences in the management of childhood diarrhea favoring males, higher asset households and urban populations would be found.

## Ethical Assurance for Protection of Human Rights

Describe in the space provided the justifications for conducting this research in human subjects. If the study needs observations on sick individuals, provide sufficient reasons for using them. Indicate how subject’s rights are protected and if there is any benefit or risk to each subject of the study.

This study addresses childhood diarrhea, a major contributor to pediatric morbidity and mortality in Bangladesh. Zinc, in the doses to be prescribed in this study, is not known to cause any major, harmful health events. It may increase the likelihood of transient vomiting. Potential, minor side effects are favorably balanced by the proof of its effectiveness in reducing the length and severity of a diarrheal episode and the decreased risk of a subsequent episode. It has also been demonstrated to reduce subsequent mortality by 50%.

Parents of children with diarrhea will be interviewed once during or shortly after an episode. Any child found to be moderately to severely dehydrated or experiencing bloody diarrhea will be referred to the nearest MOHFW or NGO health facility.

This study is not prescribing treatment, but does promote its use through a mass media campaign and provider sensitization + training. It will monitor trends in treatment practice, with a focus on zinc as a treatment. All caretakers/parents interviewed will be requested a verbal consent.

## Use of Animals

Describe in the space provided the type and species of animals that will be used in the study. Justify with reasons the use of particular animal species in the experiment and the compliance of the animal ethical guidelines for conducting the proposed procedures.

Not applicable

## Literature Cited

Identify all cited references to published literature in the text by number in parentheses. List all cited references sequentially as they appear in the text. For unpublished references, provide complete information in the text and do not include them in the list of Literature Cited. There is no page limit for this section, however exercise judgment in assessing the “standard” length.

1. Black RE. Zinc deficiency, infectious disease and mortality in the developing world. J Nutr 2003;133(5Suppl1):1485S-1489S.
2. Sazawal S, Black RE, Bhan MK, Bhandari N, Sinha A, Jalla S. Zinc supplementation in young children with acute diarrhea in India. N Engl J Med 1995;333:839-44.
3. Roy SK, Tomkins AM, Haider R, et al. Impact of zinc supplementation on subsequent growth and morbidity in Bangladeshi children with acute diarrhea. Eur J Clin Nutr 1999;53:529-34.
4. Faruque AS, Mahalanabis D, Haque SS, Fuchs G, Habte D. Double-blind, randomized, controlled trial of zinc or vitamon A supplementation in young children with acute diarrhea. Acta Paediatr 1999;88:154-60.
5. Fontaine O. Effect of zinc supplementation on clinical course of acute diarrhoea. J Health Popul Nutr 2001; 19(4):339-346.
6. Bhutta ZA, Bird SM, Black RE, Brown KH, Gardner JM, Hidayat A, et al. Therapeutic effects of oral zinc in acute and persistent diarrhea in children in developing countries: pooled analysis of randomized controlled trials. Am J Clin Nitr 2000;72:1516-22.
7. 7. Joint WHO-External consultant group. Effect of zinc supplementation on clinical course of acute diarrhea. J Health Popul Nutr 2001;19:338-46.
8. Baqui AH, Black RE, El Arifeen S, Yunus M, Chakraborty J, Ahmed S et al. Effect of zinc supplementation started during diarrhoea on morbidity and mortality in Bangladeshi children: community randomised trial. BMJ 2002; 325(7372):1059.
9. WHO/UNICEF Joint Statement. Clinical management of acute diarrhea. Geneva: World Health Organization, 2004.
10. National coverage survey of childhood diarrhea management practices in Bangladesh. Health and Science Bulletin, ICDDR,B, 2005;3(2):11-17.
11. Bennett S, Woods T, Liyanage WM, Smith DL. A simplified general method for cluster-sample surveys of health in developing countries. World Health Statist Quart 1991;44:98-106.
12. Gwatkin D, Rustein S, Johnson K, Prande R, Wagstaff A. Socio-economic differences in health, nutrition and population in Bangladesh. World Bank, 2000 (http://poverty.worldbank.org/ library/view/4212/)
13. Filmer D, Pritchard L. Estimating wealth effects without expenditure data: an application to educational enrollments in states of India. World Bank Policy Research Working Paper No. 1994. Washington DC: Development Research Group (DECRG), The World Bank, 1988.

## Dissemination and Use of Findings

Describe explicitly the plans for disseminating the accomplished results. Describe what type of publication is anticipated: working papers, internal (institutional) publication, international publications, international conferences and agencies, workshops etc. Mention if the project is linked to the Government of the People’s Republic of Bangladesh through a training programme.

The results of this study will be disseminated through peer reviewed journal publication, the Health Science Bulletin of ICDDR,B, conference presentations (national and international), workshops(national and international) and the SUZY Project technical interest group. In addition, findings will be discussed with policy makers and programmers in the public and private sectors.

## Collaborative Arrangements

Describe briefly if this study involves any scientific, administrative, fiscal, or programmatic arrangements with other national or international organizations or individuals. Indicate the nature and extent of collaboration and include a letter of agreement between the applicant or his/her organization and the collaborating organization.

None. We will work with MOHFW or City Corporation officials in the participating sites.

#

# Budget Justifications

­­

Please provide one page statement justifying the budgeted amount for each major item. Justify use of human resources, major equipment, and laboratory services.

**Personnel:**

PI: 20% time, already covered under SUZY grant

Data manager/statistician: (NOA 50%) Responsible for data quality, management and statistical analyses

Field Coordinator: (NOA FT) Responsible for implementation and conduct of surveys in all sites. This includes preparing field sites for visits.

FROs: They will supervise a team of 8 FRAs each.

FRAs: They will carry out the interviews/data collection. The # of FRAs is based upon the anticipated average # of completed interviews per week and the need to complte on survey round in 10 weeks.

Travel:

Vans, with a driver, will be rented locally if required. We will need two 12 seaters (with the exception of the Dhaka sites).

Per-diems are based upon Centre rates and an estimated 90 days in the field.

The investigators will visit the field sites on six occasions.

Rental:

To support the field sites $5,000 has been budgeted for Kamalapur, Mirsarai, Abhoynagar, Mirpur and Sylhet.

Equipment:

One desktop PC and printer will be purchased for use by the data management unit.

#

# Other Support

Describe sources, amount, duration, and grant number of all other research funding currently granted to PI or under consideration.

none for this proposed study

## Appendix 1: Voluntary Consent Form

**International Centre for Diarrhoeal Disease Research, Bangladesh**

**Voluntary Consent Form**

Title of the Research Protocol: **Monitoring the Impact of the SUZY Project Role-out of Zinc**

**as a Treatment for Childhood Diarrhea**

Principal Investigator: Charles P. Larson

Before recruiting into the study, the study subject must be informed about the objectives, procedures, and potential benefits and risks involved in the study. Details of all procedures must be provided including their risks, utility, duration, frequencies, and severity. All questions of the subject must be answered to his/ her satisfaction, indicating that the participation is purely voluntary. For children, consents must be obtained from their parents or legal guardians. The subject must indicate his/ her acceptance of participation by signing or thumb printing on this form.

**VERBAL CONSENT FORM**

________________________________________________________________________

Subject ID # [site ___ ___ / cluster ___ ___ / case # ___ ___] Interviewer ID # ___________________

[Greetings] I come from the ICDDR,B (International Centre for Diarrheal Disease Research), also known as the Cholera Hospital in Dhaka. We are carrying out a survey in many parts of Bangladesh to help us understand how parents treat their young children when they have diarrhea and how that might be changing. Your village/community has been chosen to be part of this survey.

In your village we are looking for children under five years of age who have had diarrhea in the past two weeks, such as your child. We would like to interview you (parent or caretaker) in order to know what you have done to manage this illness episode. If you can remember, we also want to know approximately how much money you spent on this most recent diarrheal illness.

If you should agree to participate in this survey, I will be asking you to answer questions about you, your family, your child’s illness and what you did to take care of your child. This will take about 20 minutes of your time. We are not recording your name. All information will remain confidential and a record of this interview will be kept in a safe, secure place. You may chose not to answer a question and you may stop the interview at any time.

We are asking for your verbal consent to complete this interview.

[ ] verbal consent not given

[ ] verbal consent given, signed by interviewer _________________________________

Date: _____ ______ _______

DD MM YY

**Appendix 2: Comments of External Reviewers**

## Appendix 3: Response to External Reviewer’s Comments

The comments and response to the external review carried out at the time of the initial RRC submission are attached.

## Appendix 4: Abstract Summary covering eight points specified by the ERC

**INFORMATION TO INCLUDE IN ABSTRACT SUMMARY**

The Committee will not consider any application which does not include an abstract summary. The abstract should summarize the purpose of the study, the methods and procedures to be used, by addressing each of the following items. If an item is not applicable, please note accordingly:

1. Describe the requirements for a subject population and explain the rationale for using in this population special groups such as children, or groups whose ability to give voluntary informed consent may be in question.

***This survey will focus on under –five diarrheal illness episodes, as this is the recommended age-group to receive zinc treatment. Informed, voluntary consent will be obtained from the parent or primary guardian.***

1. Describe and assess any potential risks – physical, psychological, social, legal or other – and assess the likelihood and seriousness of such risks. If methods of research create potential risks, describe other methods, if any, that were considered and why they will not be used.

***There are no risks we can identify that are associated with this survey*.**

1. Describe procedures for protecting against or minimizing potential risks and an assessment of their likely effectiveness.

# *None*

1. Include a description of the methods for safeguarding confidentiality or protecting anonymity.

***There is no identifying information onn the questionnaire. All questionnaires will be placed in a locked cabinet or box at the end of each day.***

1. When there are potential risks to the subject, or the privacy of the individual may be involved, the investigator is required to obtain a signed informed consent statement from the subject. For minors, informed consent must be obtained from the authorized legal guardian or parents of the subject. Describe consent procedures to be followed including how and where informed consent will be obtained.
2. If signed consent will not be obtained, explain why this requirement should be waived and provide an alternative procedure.

***We will obtain a verbal consent and require the interviewer to sign off that this was obtained. The questions to be asked are not threatening nor does this information place anyone at risk for physical, psychological or social harm.***

1. If information is to be withheld from a subject, justify this course of action.

***No information is being withheld****.*

1. If there is a potential risk to the subject or privacy of the individual is involved in any particular procedure include a statement in the consent form stating whether or not compensation and/or treatment will be available.

***No potential risks have been identified.***

1. If study involves an interview, describe where and in what context the interview will take place. State approximate length of time required for the interview.

***The interview will take place in the child’s home at a time convenient for the parent/guardian. It requires approximately 20 minutes to complete.***

1. Assess the potential benefits to be gained by the individual subject as well as the benefits which may accrue to society in general as a result of the planned work. Indicate how the benefits outweigh the risks.

***Following the interview, all unaware participants will be informed of the usefulness of zinc. From a societal standpoint, the findings of this survey guide the equitable scale up of zinc across Bangladesh.***

1. State if the activity requires the use of records (hospital, medical, birth, death or other), organs, tissues, body fluids, the fetus or the abortus.

#### *None required*

The statement to the subject should include information specified in item 2,3,4,5(c) and 7 as well as indicating the approximate time required for participation in the activity.

Check-List

**CHECK-LIST FOR SUBMISSION OF RESEARCH PROTOCOL**

**FOR CONSIDERATION OF RESEARCH REVIEW COMMITTEE (RRC)**

**[Please check (X) appropriate box]**

| 1. Has the proposal been reviewed, discussed and cleared at the Division level? | | | | | | |
| --- | --- | --- | --- | --- | --- | --- |
| Yes |  | | No | |  | |
| If No, please clarify the reasons: | | | | | | |
| 1. Has the proposal been peer-reviewed externally? | | | | | | |
| Yes |  | | No | |  | |
| If the answer is ‘No’, please explain the reasons: | | | | | | |
| If yes, have the external reviews’ comments and their responses been attached | | | | | | |
| Yes | |  | | No | |  |
| 1. Has the budget been cleared by Finance Department? | | | | | | |
| Yes |  | | No | |  | |
| If the answer is ‘No’, reasons thereof be indicated: | | | | | | |
| 1. Does the study involve any procedure employing hazardous materials, or equipments? | | | | | | |
| Yes |  | | No | |  | |
| If ‘Yes’, fill the necessary form. | | | | | | |
| ______________ _________  Signature of the Principal Investigator Date | | | | | | |
